# Monitoring Urban Zoonotic Virus Activity: Are City Rats a Promising Surveillance Tool for Emerging Viruses?

**DOI:** 10.3390/v14071516

**Published:** 2022-07-11

**Authors:** Jeremy V. Camp, Amélie Desvars-Larrive, Norbert Nowotny, Chris Walzer

**Affiliations:** 1 Institute of Virology, University of Veterinary Medicine Vienna, 1210 Vienna, Austria; norbert.nowotny@vetmeduni.ac.at; 2 Center for Virology, Medical University of Vienna, 1090 Vienna, Austria; 3 Institute of Food Safety, Food Technology and Veterinary Public Health, University of Veterinary Medicine Vienna, 1210 Vienna, Austria; amelie.desvars@vetmeduni.ac.at; 4 Complexity Science Hub Vienna, 1080 Vienna, Austria; 5 VetFarm, University of Veterinary Medicine Vienna, 1210 Vienna, Austria; 6 Department of Basic Medical Sciences, College of Medicine, Mohammed Bin Rashid University of Medicine and Health Sciences, Dubai P.O. Box 505055, United Arab Emirates; 7 Research Institute of Wildlife Ecology, University of Veterinary Medicine Vienna, 1210 Vienna, Austria; chris.walzer@vetmeduni.ac.at; 8 Wildlife Conservation Society, Global Conservation Program, Bronx, NY 10460, USA

**Keywords:** rats, urban, emerging infectious diseases, viral zoonoses, environmental monitoring, disease surveillance

## Abstract

Urban environments represent unique ecosystems where dense human populations may come into contact with wildlife species, some of which are established or potential reservoirs for zoonotic pathogens that cause human diseases. Finding practical ways to monitor the presence and/or abundance of zoonotic pathogens is important to estimate the risk of spillover to humans in cities. As brown rats (*Rattus norvegicus*) are ubiquitous in urban habitats, and are hosts of several zoonotic viruses, we conducted longitudinal sampling of brown rats in Vienna, Austria, a large population center in Central Europe. We investigated rat tissues for the presence of several zoonotic viruses, including flaviviruses, hantaviruses, coronaviruses, poxviruses, hepatitis E virus, encephalomyocarditis virus, and influenza A virus. Although we found no evidence of active infections (all were negative for viral nucleic acids) among 96 rats captured between 2016 and 2018, our study supports the findings of others, suggesting that monitoring urban rats may be an efficient way to estimate the activity of zoonotic viruses in urban environments.

## 1. Introduction

During the last decades, spillover of viruses from wildlife hosts have caused high-impact diseases in humans, e.g., hemorrhagic fevers caused by hantaviruses or arenaviruses, two epidemics of severe acute respiratory syndrome related to zoonotic-origin coronaviruses (SARS-CoV), Ebola virus disease, and influenza A. Anthropogenic changes, including increasing human population density, increasing international travel, land-use change, and urban sprawl, appear to be drivers in the spillover and spread of zoonotic viruses to humans [1,2]. In particular, cities are unique ecosystems where dense human populations and their companion animals live in relatively close proximity with wildlife species.

Given that the majority (60.3%) of emerging infectious diseases are caused by zoonotic pathogens, of which 71.8% originate from wildlife [3], knowledge of zoonotic pathogens carried by wildlife hosts is critical to understanding pathogen prevalence in the environment, geographic distribution, and risk of spillover to humans.

Brown rats (*Rattus norvegicus*) are described as “urban exploiters” [4] in that they proliferate in urban settings where they live in close proximity with humans [5]. As urban brown rats are hosts of several zoonotic pathogens [6,7], they may act as reservoirs of these pathogens to humans and livestock [8,9]. However, information on the viruses carried by urban wild rats that could pose a zoonotic risk to human health is scarce.

The objective of this study was to conduct a cross-sectional survey to assess the prevalence of some viruses of zoonotic importance in wild brown rats within the city center of Vienna, Austria. The studied sites are highly frequented by humans and were suspected to present a high rat density. We focused our investigation on a limited number of viruses, previously identified in rodents at different locations worldwide [9,10,11] based on three criteria:

1. The virus was previously reported in urban brown rats, e.g., Seoul orthohantavirus (SEOV) [12,13], hepatitis E virus (*Orthohepevirus A*, HEV) [14,15,16], influenza A virus (IAV) [17], and coronaviruses [18,19,20];
2. The virus is known to circulate in Austria, i.e., West Nile virus (WNV) [21,22], Usutu virus (USUV) [21,23,24], HEV, which was recently detected in urban rats in Vienna [25], Puumala and Tula orthohantaviruses [26,27,28,29], or neighboring regions, i.e., Dobrava-Belgrade orthohantavirus was reported in urban rats in neighboring Hungary [30] and orthopoxviruses (cowpox virus), diagnosed in free-roaming Austrian cats most likely following rodent exposure [31], including zoonotic transmission to humans through direct contact with the poxvirus lesions of the cat [32];
3. Rodents are thought to be the natural reservoir for the virus, i.e., encephalomyocarditis virus (*Cardiovirus A*, EMCV) [33].

These viruses are transmitted from rat to rat mainly via direct or indirect contact with excreta (e.g., hantaviruses, encephalomyocarditis virus) or saliva (e.g., hantaviruses, coronaviruses), perhaps during intraspecific aggression (e.g., hantaviruses) [34]. Some viruses are considered non-seasonal in rats (e.g., hantaviruses [11,35]), while others have demonstrated seasonal variations (e.g., for seasonal IAV [17]) in rat populations. Because urban brown rats are synanthropic and may come into contact with food supplies [5,36,37], rat-to-human transmission is likely to occur via direct or indirect contact with rat excreta (e.g., hantaviruses, HEV, encephalomyocarditis virus) or saliva (e.g., hantaviruses), via the bite of competent vector (e.g., WNV, USUV), or direct inoculation via close contact with infected animals (poxviruses). In general, much is unknown about the role of urban rats in the transmission of zoonotic viruses to humans, and studies such as this one may shed light on poorly understood aspects of viral zoonoses (e.g., precise transmission routes, seasonality, etc) [1,38,39].

Eight zoonotic viruses from seven virus taxa were ultimately included in the study: flaviviruses (specifically WNV and USUV), Old World hantaviruses (specifically Seoul orthohantavirus), HEV, coronaviruses (CoVs), IAV, EMCV, and poxviruses (subfamily *Chordopoxvirinae*). We chose not to investigate the potential presence of other rat-borne viruses with poorly substantiated zoonotic potential (e.g., astroviruses, noroviruses), although these have been documented in wild brown rats, including in neighboring countries [40,41].

## 2. Materials and Methods

### 2.1. Ethical Statement

This study followed institutional and national standards for the care and use of animals in research. It was approved by the institutional ethics and animal welfare committee and the national authority (GZ 68.205/0196-WF/V/3b/2016).

### 2.2. Study Areas and Sampling Methods

*Rattus norvegicus* were trapped between 12 September 2016 and 13 November 2018 in three sites highly frequented by humans in the city center of Vienna, Austria: (i) at a promenade along the Danube canal (mean coordinates of the trapped rats in decimal degrees: 16.365 N, 48.226 E); (ii) at Karlsplatz (16.363 N, 48.200 E), one of the tourist attractions in the city; and (iii) at Schwedenplatz (16.375 N, 48.212 E), a cruise ship port on the Danube river. These sites were chosen as rats could be observed during daytime, suggesting that the rats were abundant and that these locations may represent critical interfaces for virus transmission between rats and humans. Rats were captured live in spring and autumn season (we avoided too cold/warm temperatures due to ethical and animal welfare considerations) using Manufrance live-traps (280 × 100 × 100 mm). Traps were set between 17.00 and 19.30 and retrieved the following morning between 6.00 and 8.00 (more details on trapping can be found in [42,43]). Live captured animals were transferred to a pathology laboratory where they were anesthetised in an induction chamber using 5% isoflurane before euthanasia via an intra-peritoneal barbiturate overdose. Rats were identified to the species level based on morphological characters. For each animal, morphological data were recorded. We chose to sample and analyze the lung tissue because it is the preferential organ for detection of hantaviruses [44], EMCV [45], and influenza A viruses [17]. It is also the most highly vascularized organs in rats [46], enabling us to potentially monitor blood-circulating viruses even if they do not have a lung tropism. During necropsy, lung tissue was collected aseptically and placed into RNAlater™ (ThermoFisher, Waltham, MA, USA). A ten mm tail tip was sampled for molecular barcoding purposes. All samples were maintained at −80 °C until RNA or DNA extraction.

### 2.3. Barcoding

To confirm the morphological identification of the *Rattus* species, we followed the DNA barcoding protocol as described in [47], consisting in the amplification and sequencing of a 585-bp fragment of the mitochondrial DNA (mtDNA) D-loop.

### 2.4. Preparation of Tissue

Lung tissue was removed from RNAlater, and an approximately 1 g section was placed into 400 µL ice-cold sterile phosphate-buffered saline (PBS) in a tube with 4 copper-coated steel beads. The tissue was homogenized on a TissueLyser (Qiagen, Hilden, Germany) for 3 min at 30 Hz and then centrifuged at 8000× *g* for 4 min at 4 °C. Nucleic acids were extracted from 200 µL of cleared homogenate with a commercial kit (ZymoResearch, Irvine, CA, USA). Nucleic acid extraction was checked by spectrophotometry (NanoVue, Biochrom GmbH, Berlin, Germany) and confirmed that extraction was successful.

### 2.5. Detection of Viral Nucleic Acids

Virus nucleic acids were detected by following various previously published PCR protocols using 2.5–5 µL of nucleic acid template (Table 1). Highly sensitive real time reverse transcription PCR (RT-qPCR) to detect RNA viruses was preferred when protocols were available using Luna^®^ One-step RT-qPCR mix (New England Biolabs, Ipswich, MA, USA) on an Applied Biosystems 7500 light cycler with published temperature cycling programs (Table 1). Conventional RT-PCR to detect RNA viruses was performed using a one-step RT-PCR mix (One*Taq*^®^, New England Biolabs) followed by capillary gel electrophoresis (QIAxcel, Qiagen, Hilden, Germany) to visualize amplicons. Similarly, conventional PCR was used to detect poxviruses using Go*Taq* G2 mix (Promega, Madison, WI, USA) followed by capillary gel electrophoresis to visualize amplicons. Positive controls were used for flaviruses (a WNV lineage 4c isolate [48] and an USUV cell culture isolate “939/01” [24]), as these are well-characterized in our laboratory and potential false positives could be identified by sequencing. Otherwise, no positive controls were used to reduce the possibility of false-positive results. Instead, we used common diagnostic assays to detect some viruses (coronaviruses, hantaviruses, influenza A virus, and chordopox viruses) or used multiple tests (one RT-qPCR and one RT-PCR for hepatitis E virus; two RT-qPCR and one nested RT-PCR for EMCV) to reduce the possibility of false negatives. The samples were screened for the following viruses: CoVs [49,50], flaviviruses [51] including specific assays to detect WNV [24] or USUV [52], Old World hantaviruses [53], two assays to detect HEV [54,55], IAV [56], three assays to detect EMCV [57,58,59], and poxviruses [60] (Table 1).

## 3. Results

### 3.1. Trapping

Ninety-six animals were captured, all identified as *R. norvegicus* (Danube canal: 44; Karlsplatz: 45; Schwedenplatz: 7). The generated barcode sequences confirmed the morphological identification. Among the captured rats, 50 (52.1%) were males (Danube canal: 24; Karlsplatz: 23; Schwedenplatz: 3). The median body mass and length (nose tip to anus) were 157.9 g and 188 mm for rats caught at Danube Canal, 134.2 g and 180.1 mm at Karlsplatz, and 58 g and 135 mm at Schwedenplatz, respectively (Table S1), indicating that the sampled animals were relatively young [61].

### 3.2. Detection of Viruses

No virus nucleic acids were detected in the rat lung tissue samples. For viruses which we presumed were highly likely to be detected, multiple assays were performed and were all negative: hepatitis E virus (one RT-qPCR and one RT-PCR) and EMCV (two RT-qPCR and one nested RT-PCR) (Table 1).

## 4. Discussion

Among the zoonotic viruses investigated here, two, namely EMCV and HEV, were the most likely candidates for detection in urban brown rats. Rodents are reservoirs and vectors of EMCV, and sporadic outbreaks in domestic animals have been linked to rodent exposure [62]. In Italy, outbreaks within zoos or on farms have occasionally been associated with either EMCV-positive rodents [58] or increased rodent abundance [61]. EMCV is present in Austria and neighboring countries, and exposure has been detected in domestic pigs, where the virus causes little to no clinical pathology [63,64], and in humans [65,66]. Specifically, human exposure was linked to hunters and zoo workers, thus contact to wild game animals and captive wildlife may be risk factors for exposure in Austria.

EMCV can be detected up to 22 dpi in the lungs of experimentally inoculated laboratory rats with a high rate of transmission between rats (R_0_ >> 1) [45], nonetheless the infection is ultimately transient. Therefore, we cannot rule out a low level of EMCV infection in the urban rat population; however, a larger sample size may be necessary to detect active virus infections.

We also expected to detect HEV in our survey of urban brown rats, particularly as HEV has recently been detected in urban brown rats captured in Vienna [25]. Recent virological and serological surveys of Austrian blood donors suggested that approximately 14% of the human population had been exposed to HEV, and 0.01% had active infections [67,68]. HEV (specifically the species *Orthohepevirus A*) is an emerging viral zoonosis, and in Europe, wild ungulates (wild boar and deer) and domestic pigs are the principal reservoirs [69]. HEV-positive wild *R. norvegicus* were detected in the cities of Lyon, France (12/81, 15%) [70]; Hamburg, Germany (2/30, 6.7%) [15]; and Vienna, Austria (7/43, 16.2%) [25], although in these studies, the virus was detected in liver [25,70] and feces [15,25].

Studies in Germany have revealed that HEV isolates from brown rats were phylogenetically different from epizootic strains [14] and have since been assigned to *Orthohepevirus C* species (genotype C1). The so-called “rat HEV” was first detected with a “broad-spectrum RT-PCR” [15], which was not used in our study. In the previous study that detected HEV RNA-positive rats in Vienna, all were *Orthohepevirus C* (i.e., “rat HEV”), and not the epizootic *Orthohepevirus A*, which has been isolated from a variety of animals and linked to human disease [25]. Importantly, in that study, no orthohepeviruses were detected by two RT-qPCR assays (one of which was specific to epizootic HEV and was used in our study) but were rather detected by a conventional RT-PCR assay (also used in our study) [25]. Thus, although we did not use the “broad-spectrum” HEV assay [15], Ryll et al. clearly demonstrated that rat HEV is circulating in Vienna and could be detected by the conventional RT-PCR used in our study [25]. This may suggest the distribution of HEV in the urban rat population in Vienna is spatially focal and/or seasonal, as has been observed elsewhere [14].

The fact that we sampled lung tissue (and not liver, feces, or other tissue) should not have been a major limitation, as HEV and EMCV are blood-borne pathogens and therefore present in the large blood-volume of the rodent lung. Overall serological testing combined with molecular testing would provide more information regarding the exposure of urban rats to zoonotic viruses and the zoonotic risk to humans and domestic animals. Serological testing is needed as not all the viruses cause lifelong infection in rodents, and infection is not necessarily concomitant with the presence of antibodies (e.g., [14]). From our results, we can only infer that the rate of active viral infection of urban rats at the three investigated sites is low, but we did not determine their exposure to these viruses at these sites.

Among the other viruses that were screened, hantaviruses are blood-borne viruses which are present in Austria but were not detected in our study. In Austria, Puumala orthohantavirus is the most common hantavirus (the reservoir host is the bank vole, *Myodes glareolus*), causing the mild disease “nephropathia epidemica” in humans [26,27,28]. The more pathogenic Dobrava-Belgrade orthohantavirus (the reservoir host is the yellow-necked mouse, *Apodemus flavicollis*) is also present and may cause a more severe form of disease termed hemorrhagic fever with renal syndrome (HFRS) [71,72]. Evidence of cross-species transmission of rodent-borne hantaviruses exists [73,74,75]; although we note that *Apodemus* sp. were occasionally found in the traps, we cannot infer the likelihood of virus spillover. The detection of SEOV may have been more likely, as black rats (*Rattus rattus*) and brown rats are known reservoirs. SEOV may also cause HFRS in humans and has a wide geographic distribution due to global trade: SEOV RNA was detected in the lungs of wild urban brown rats in France [76,77], Belgium [44], UK [78], and New York City, USA [9]. Hantavirus reservoirs are typically persistently infected, and therefore it is likely that we would have detected infection if present. Therefore, our data support the hypothesis that human exposure to hantaviruses is unlikely in urban habitats of Vienna, and risk of spillover of endemic hantaviruses to other rodents is also limited.

The mosquito-borne flaviviruses WNV and USUV are endemic in Austria, and are known to cause occasional disease in humans, birds, and horses [22,79]. These zoonotic viruses are maintained in an enzootic cycle involving *Culex* mosquitoes and avian hosts. USUV and WNV have never been reported in wild *R. norvegicus*, although antibodies to WNV were detected in *R. rattus* and/or *R. norvegicus* in Pakistan, Israel, Austria, Tunisia, central Africa, and Madagascar, as well as Maryland, Washington, DC, and Louisiana, USA [80]. USUV has been detected in wild *R. rattus* and other rodents in Senegal [81]. As 2018 was notable for an extraordinarily high rate of WNV infection in humans, mosquitoes, and horses [82], transmission to other urban mammals during this time appeared probable; however, we detected no USUV or WNV in our samples. We used robust universal flavivirus primers as well as virus specific USUV and WNV primers, which are well documented to amplify many flavivirus species [51]; however, we cannot exclude the fact that rats, like other mammals, are dead-end hosts for these arboviruses with a brief stage of low viremia.

Influenza A virus is maintained in aquatic avian cycles and enters human epidemic transmission cycles via domestic swine or can spill-over to humans directly [83]. However, IAV was described in the lung of urban brown rats in Boston, USA (2/163, 1.2%) [17]. Rats are unlikely to be important hosts of IAV. Similarly, to our knowledge, rodent-human transmission of coronaviruses has never been recorded, although human coronavirus OC43 is thought to share a common ancestor with some rodent coronaviruses [84]. Murine coronaviruses (genus *Betacoronavirus*), including murine hepatitis virus and sialodacryoadenitis virus, can be common in laboratory rodents, including *R. norvegicus* strains, and many species/strains of related coronaviruses have been characterized from wild rat populations [10,19,20,84,85]. Human infection with zoonotic coronaviruses is well documented (e.g., SARS-CoV-1, SARS-CoV-2, MERS-CoV), but rats have never been conclusively implicated in the transmission cycle [20,86]. Therefore, the lack of IAV and CoV in our samples is not surprising. We recommend that serological tests or virological tests of other tissue samples (e.g., intestine or feces) are investigated in a future surveillance project of urban rats.

Finally, poxviruses are known to be transmitted from rats to humans and domestic animals. According to previous surveys, cowpox is present in several rodent species in Central Europe with a high seroprevalence [87,88]. While these broad surveys of wild rodents did not include rats, exposure to rats has been suspected in some confirmed human poxvirus infections: cowpox virus was detected in a patient with an exposure to a pet rat [89]; poxvirus was isolated from skin lesions of a wild *R. norvegicus* in Kuwait [90]; and in the Netherlands, a cowpox virus was isolated from wild *R. norvegicus* [91,92], including a case of direct transmission to a human [92]. Cowpox has occasionally been detected in Austria [31,32], yet cases in individuals from younger generations who did not receive the vaccinia vaccine are increasing. Future surveillance efforts should focus on serosurveillance to determine exposure to cowpox virus in the urban rat population to better ascertain the zoonotic risk of infection.

While we are confident in the diagnostic techniques used here, it is clear that detecting active infections require a larger sample size, particularly when there is a bias towards sampling a young population as expected with live traps (in contrast to kill traps, as were probably used in [25]). Calculating a sample size based on prevalence found in the literature encounters two major issues: (i) prevalence of rat-borne diseases varies spatially at the global and local scale [6,14,93]; (ii) researchers acknowledge certain challenges in studying urban rats, especially as trapping success is generally quite low [94,95], which often precludes reaching the targeted sample size. We acknowledge other potential study limitations, including the variability in sensitivity of the assays to detect a given virus and the investigation of a single tissue (versus virus-specific target tissues).

As the likelihood of infection with viruses increases with age for many viruses, and many viral infections follow an acute course, there was a low probability of detecting active virus infections [9,10]. However, zoonotic infections were detected in the examined sample in previous studies [42,43,96], while viral infections have been detected in comparable [9,35,70,76] or lower [15,25,77] sample sizes, demonstrating that a modest sample size of the rat population enables to reveal current viral infections. Therefore, targeted surveillance of the rat population for zoonotic viral pathogens should focus on older rats, or at least attempt to include all age classes. Thus, the principal limitations of our study were that few sites were investigated, relatively low sample size (n = 96), and the overall young age of the captured rats. A higher diversity of microenvironments could have revealed variations in the prevalence, therefore highlighting favorable environments for virus transmission. A bigger sample size would have provided a more accurate picture of the epizootiologic situation.

Negative results are rarely published [97]. However, the (sole) publication of positive results greatly limits a realistic perspective of the entire epidemiological situation [98]. In particular, publishing negative results helps to interpret positive results that may be obtained in the future at this location or in similar studies at different locations. Furthermore, when searching for potential animal reservoirs or vectors of (emerging) infectious diseases, having positive results only (presence data) induces a bias and restricts the perception and the understanding of the global epidemiology of these infectious diseases [99]. Publication bias (toward positive results) makes scientific literature unrepresentative of the research field. It may lead to misconception of the reality, e.g., that prevalence of zoonotic pathogen infections in urban rats is generally high. Absence data are as critical as presence data to the understanding of the eco-epidemiology of zoonotic viruses, mapping of their true geographic coverage, and assessment of contributing factors of virus emergence. Publication of absence data is of high interest for addressing the epidemiological situation at a given instance and enables dating of pathogen emergence or shift in the epidemiological situation.

In addition to eradication efforts, monitoring of wild rats for potential zoonotic viruses is potentially a valuable resource to predict future human outbreaks. For example, the detection of *Leptospira* spp. in rats has proven to be a good spatial predictor of human leptospirosis cases in leptospirosis-endemic urban habitats [100]. Moreover, urban brown rats could theoretically be used as sentinels for fine-scale spatial monitoring of environmental contamination with antimicrobial resistant bacteria [42], lead [101], and other heavy metals [102]. Therefore, within a One Health approach and operationalization, (sero)surveillance of rat populations may prove valuable to assessing the zoonotic risk from viruses, particularly of EMCV or HEV, in the human population but also in domestic animals, in urban and periurban habitats. The use of urban brown rats as sentinels for the active surveillance of some targeted viral pathogens should be further evaluated as a promising surveillance tool in settings where the viruses are known as well as not yet recorded.

## 5. Conclusions

Our transverse study provided absence data about eight zoonotic viruses in wild urban brown rats sampled across three sites within the city center of Vienna, Austria, that are highly frequented by humans. Our findings showed that in these specific sites and at the time of sampling, rats did not constitute a hazard for the zoonotic transmission of the investigated viruses to humans. We recommend the publication of absence data (in any format, short communication, dataset, dedicated website) about rat-borne pathogens for better, unbiased assessments of emergence risk factors.

## Figures and Tables

**Table 1:** PCR-based methods to detect various zoonotic viruses in captured rats.

Virus Family	Target Virus/Taxon	Assay	Primers/Probes	Ref.
*Coronaviridae*	Coronaviruses	RT-PCR	Fwd primer from [49], PanCoV-13-RV [50]	[49,50]
*Flaviviridae*	Flavivirus (universal)	RT-qPCR	PF1S, PF2Rbis/SYBR Green	[51]
	West Nile virus	RT-PCR	WNV-10090f, WNV-10807r	[22]
	Usutu virus	RT-PCR	Usu9170f, Usu9704r	[52]
*Hantaviridae*	Hantaviruses	RT-PCR, nested	HAN-L-F1, HAN-L-R1; nested HAN-L-F2, nested HAN-LR2	[53]
*Hepeviridae*	Hepatitis E virus	RT-qPCR	JVHEVF, JVHEVR/JVHEVP	[54]
	Hepatitis E virus	RT-PCR	rHEV-SW-for, rHEV-SW-rev	[55]
*Orthomyxoviridae*	Influenza A virus	RT-qPCR	FLUAM-1F, FLUAM-1R/FLUAM-1P	[56]
*Picornaviridae*	Encephalomyo-carditis virus	RT-qPCR	5NTR-F, 5NTR-R/5NTR-P;	[59]
		RT-qPCR	2B-F, 2B-R/2B-P	[59]
	Encephalomyo-carditis virus	RT-PCR, nested	EMCVff2,EMCVrev1; nested EMCVffint3, EMCVrevint3	[57]
*Poxviridae*	*Chordopoxivirinae*	PCR	“Low GC” primers	[60]

## Data Availability

All relevant data are presented in the text of the manuscript.

## Supplementary Materials

The following supporting information can be downloaded at: https://www.mdpi.com/article/10.3390/v14071516/s1. Table S1. Dataset: characteristics of the rats (*Rattus norvegicus*) captured for this study, Vienna, Austria, 12 September 2016–13 November 2018.
